# Re-Rolling Treatment in the Fermentation Process Improves the Aroma Quality of Black Tea

**DOI:** 10.3390/foods12193702

**Published:** 2023-10-09

**Authors:** Qincao Chen, Penghui Yu, Ziyi Li, Yuhang Wang, Yafang Liu, Yin Zhu, Haihui Fu

**Affiliations:** 1 College of Agriculture, Jiangxi Agricultural University, No. 1101 Zhimin Avenue, Nanchang 330045, China; chenqincao@jxau.edu.cn (Q.C.); 18931077210@163.com (Z.L.); carti18942330763@163.com (Y.W.); lyf2805101190@163.com (Y.L.); 2 Tea Research Institute, Hunan Academy of Agricultural Sciences, No. 702 Yuanda 2nd Road, Changsha 410125, China; hncys-yupenghui@hunaas.cn; 3 Tea Research Institute, Chinese Academy of Agricultural Sciences, No. 9 Meiling South Road, Hangzhou 310008, China

**Keywords:** black tea, aroma, re-rolling, GC-MS, fermentation

## Abstract

Aroma is a vital factor influencing tea quality and value. It is a challenge to produce a kind of black tea with a floral/fruity aroma, good taste, and without a green/grassy odor simultaneously using small- and medium-leaf tea species. In this study, the effect of re-rolling treatment on the aroma quality of small-leaf Congou black tea was investigated using the methods of the equivalent quantification of aroma and gas chromatography–mass spectrometry (GC-MS). Sensory evaluation showed that re-rolling treatment improved the aroma quality of Congou black tea by conferring upon it floral and fruity scents. In total, 179 volatile compounds were identified using GC-MS, of which 97 volatiles showed statistical differences (Tukey s-b(K), *p* < 0.05). Re-rolling treatment significantly reduced the levels of alcoholic fatty acid-derived volatiles (FADVs) and volatile terpenoid (VTs), but increased the levels of aldehydic and ester FADVs, most amino acid-derived volatiles (AADVs), carotenoid-derived volatiles (CDVs), alkene VTs, and some other important volatile compounds. Based on the odor characteristics and fold changes of differential volatile compounds, hexanoic acid, hexyl formate, cis-3-hexenyl hexanoate, (Z)-3-hexenyl benzoate, hexyl hexanoate, phenylacetaldehyde, benzyl alcohol, β-ionone, α-ionone, dihydroactinidiolide, ipsenone, β-farnesene, β-octalactone, melonal, etc., were considered as the potential key odorants responsible for the floral and fruity scents of re-rolled black tea. In summary, this study provides a novel and simple processing technology to improve the aroma quality of small-leaf Congou black tea, and the results are beneficial to enriching tea aroma chemistry.

## 1. Introduction

Black tea is the most popular tea in the world due to its fascinating aroma and taste, accounting for approximately 75% of tea consumption [1]. According to the manufacturing methods, there are two main classes of black tea: Congou and CTC (crush, tear, and curl) black teas. Congou black tea is the traditional Chinese black tea, processed for about 400 years. Based on the size of the tea leaf, Congou black teas are further divided into small-, medium-, and large-leaf types in China [2].

Aroma, as a balance of numerous complex volatile compounds, is one of the key factors influencing the quality and value of teas. Small- and medium-leaf Congou black teas, for instance, Ning black tea (short name of Ningzhou Congou black tea), generally present a sweet aroma [3], while large-leaf Congou black teas, for instance, Yingde black tea, easily display a floral and fruity aroma [4]. To date, more than 700 volatile compounds have been found in teas [5]. Volatile compounds can be divided into fatty acid-derived volatiles (FADVs), amino acid-derived volatiles (AADVs), volatile terpenoids (VTs), and carotenoid-derived volatiles (CDVs) [6]. They are dramatically affected by processing technology. The basic manufacturing steps of Congou black tea include withering, rolling, fermenting, first drying, and final firing [2]. Among them, fermenting is the key step in black tea aroma and taste formation.

During this process, fatty acids, amino acids, carotenoids, and glycosidically bound volatile compounds (GBVs) are drastically decomposed into volatile compounds [7,8], which have laid the foundation for the transformation from a grassy to a sweet odor for black tea aroma [8]. In addition, other processing steps also have a notable impact on black tea aroma formation [9]. To date, many studies have been conducted to explore the influence of processing methods and conditions on black tea aroma quality, such as different withering methods [10,11], light irradiation during withering [12,13], rolling temperature [14], fermentation time [2], adding β-glucosidase during fermentation [15], and varying the temperatures and times of final firing [16].

Sweet is the typical and most common aroma for small- and medium-leaf Congou black tea, while floral and fruity aromas make small- and medium-leaf Congou black tea more attractive and thus provide better aroma quality and a higher sale price. In practical production, for small- and medium-leaf tea species, light fermentation can more easily confer on black tea floral and fruity aromas, while it also have a greater possibility to synchronously give black tea a green/grassy odor as well as a bitter and astringent taste [17]. As fermentation time is prolonged, the green/grassy odor as well as bitter and astringent taste will usually disappear, while black tea generally only presents a sweet odor without floral and fruity scents [17,18]. It is a challenge to simultaneously produce a kind of small- and medium- leaf Congou black tea with a floral/fruity aroma, good taste, and without a green/grassy odor. In our previous study, we found that the intensity of the green/grassy scent presented a notable decrease; the intensity of floral and fruity scents presented a trend of first rising, and then, falling; and the intensity of the sweet scent presented a notable increase during the fermenting step [8]. Inspired by this, we wonder whether re-rolling treatment in the fermentation process would enhance the floral and fruity scents of fermented tea leaves and thus ultimately give black tea a floral/fruity aroma. In our pre-study, this speculation was proven to be correct.

Ning black tea is a famous and time-honored small-leaf Congou black tea produced in Jiangxi Province, China [3]. In this study, Ning black tea was used as the research material to evaluate the effect of re-rolling treatment on the aroma quality of Congou black tea via the methods of the equivalent quantification of aroma and gas chromatography–mass spectrometry (GC-MS).

## 2. Materials and Methods

### 2.1. Chemicals and Reagents

Deionized water was produced using a Milli-Q water purification system (Millipore, Billerica, MA, USA). N-alkanes (C7–C40) and 1-hexanol were obtained from the J&K Scientific Corporation (Beijing, China). (Z)-3-Hexen-1-ol, linalool, cis-linalool oxide (furanoid), trans-linalool oxide (furanoid), cis-linalool oxide (pyranoid), trans-linalool oxide (pyranoid), (E)-2-nonenal, α-ionone, β-ionone, cis-3-hexenyl isovalerate, and cis-3-hexenyl hexanoate were purchased from the TCI Corporation (Tokyo, Japan). Phenylacetaldehyde and 1-octen-3-one were obtained from the Alfa Aesar Corporation (Shanghai, China). 1-Octen-3-ol, β-farnesene, geraniol, nerolidol, (E)-2-hexenal, hexanal, octanal, (E,E)-2,4-heptadienal, melonal, (E)-2-octenal, 2,2,6-trimethylcyclohexanone, 3-octen-2-one, dihydroactinidiolide, methyl salicylate, hexyl hexanoate, and 1-ethyl-1H-pyrrole-2-carboxaldehyde were obtained from the Aladdin Corporation (Shanghai, China). (E,E)-2,4-Hexadienal, β-damascenone, and β-octalactone were purchased from the Sigma-Aldrich Corporation (Shanghai, China).

### 2.2. Manufacturing of Black Tea Samples

**Figure 1:**
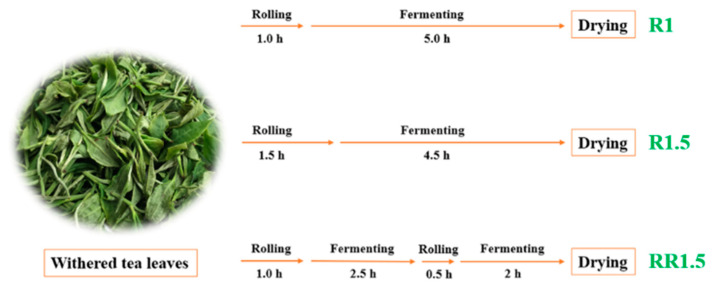
The rolling and fermenting procedures of the three black teas.

The clonal Ningzhou No. 2 (*Camellia sinensis* (L) O. Kuntze) tea plant, which was planted in the tea garden of Jiangxi Tonggu Tea Co., Ltd (Yichun, China), was selected for our study. Approximately 25 kg fresh leaves (one bud and two leaves) were evenly spread for indoor natural withering. As the moisture content reached about 62.0%, the tea leaves were rolled using a roller (6CR-10, Fuyang Machinery Co., Ltd., Hangzhou, China) and fermented at 32 °C with 90% humidity in an environmentally controlled cabinet (PRX-450D, Saifu Machinery, Suzhou, China). The rolling and fermenting durations for different batches were as follows: R1 underwent 1 h of rolling followed by 5 h of fermentation, R1.5 went through 1.5 h of rolling followed by 4.5 h of fermentation, and RR1.5 underwent a sequence of 1 h rolling, 2.5 h fermentation, 0.5 h rolling, and 2 h fermentation (Figure 1). The total rolling and fermentation time for all three samples was 6 h (Figure 1). After fermenting, the tea leaves were first dried at 110 °C for 20 min, and then, cooled for 30 min. Finally, the tea leaves were dried at 85 °C for 15 min to obtain black tea samples. These tea samples were stored at −20 °C and were ground into a homogeneous powder using a mill (IKA Werke GmbH, Staufen, Germany), as in prior and further analyses.

### 2.3. Equivalent Quantification of Aroma

Sweet scent is the typical aroma for black tea and is formed in the fermenting process [8]. Black teas may also present floral and fruity scents owing to their varietal specificity and processing methods [16,19], or retain green/grassy scents [4,11]. Thus, to better appraise the aroma quality, the aromas of three black teas were evaluated via the method of the “equivalent quantification of aroma” referred to in our previous study [8]. In this method, the scents were categorized as sweet, floral and fruity, and green scents. The evaluation group consisted of 5 experienced tea assessors (two males and three females), and they were randomly divided into two groups to independently evaluate the aroma of the tea samples. Tea brewing was conducted with adherence to the Chinese national standard of the “Methodology of sensory evaluation of tea” (GB/T 23376-2018). Briefly, 3.0 g non-powdered teas were brewed with 150 mL of boiled water in a special judging cup. After brewing for 5.0 min, the tea infusions were then transferred to a bowl. Afterwards, the infused tea samples were sniffed to evaluate the aroma characteristics and intensity. The black tea samples were brewed twice for two groups to separately evaluate the aroma. The aroma was divided into three characteristics: green, sweet, and floral/fruity scent. The aroma intensities were scored from 1 to 4, as follows: 1, weak; 2, moderate; 3, strong; and 4, extremely strong. The average scores determined by the assessors represented the final intensity.

### 2.4. Determination of Volatile Compounds

Volatile compounds were extracted via the method of headspace solid-phase microextraction (HS-SPME) referred to in our previous study [6]. Triplicate powdered samples (1.0 g) were first each placed in 20 mL glass vials (O.D. × H 22.5 × 75.5 mm; Supelco, Bellefonte, PA, USA), and then, we added 6 mL boiling deionized water; the vials were immediately sealed with a cover and equilibrated for 3.0 min at 60 °C in a heating oscillator; after that, a carboxen/polydimethylsiloxane coating fiber (75 µm; 1 cm; Supelco, Inc., Bellefonte, PA, USA) was used to adsorb volatile compounds for 60 min; finally, the volatile compounds were subsequently desorbed at 250 °C for 5 min for GC-MS analysis.

GC–MS analysis was carried out using a GC system (8860, Agilent Technologies, Santa Clara, CA, USA) coupled with an MS detector (5977B, Agilent Technologies, Santa Clara, CA, USA). The column was a HP-5MS column (30 m × 250 µm × 0.25 µm, Agilent Technologies; Santa Clara, CA, USA), and the carrier gas was helium (99.999%) with a constant flow of 1.6 mL/min. The GC rising temperature procedure was as follows: 40 °C held for 5 min, slowly increasing to 160 °C at 3 °C/min, and then, increasing to 250 °C at 15 °C/min, which was held for 5 min. The splitless injection mode was applied.

The MS conditions were as follows: the ionization mode was EI; the ionization voltage was 1228 V; the ion source temperature was 230 °C; the quadrupole temperature was 150 °C; a full scan mode was used with a mass scan of 33–600 u; the electron impact ionization was –70 eV.

The raw data were processed using LECO Chroma TOF software (V 4.51.6.0). The parameters for processing individual sample data were as follows: the minimum S/N was set at 20, and the minimum similarity match was set at 750 (maximum of 1000); the 1st D peak width was 25. The integrated peak table of all samples was accomplished using the statistical comparison function in the Chroma TOF software (V 4.51.6.0)as well, and the deviation in the 1st D retention time (RT) was 0.2 min. The Kovats retention index (RI) of volatile compounds was automatically calculated using a series of n-alkanes (C7–C40) via the LECO Chroma TOF software (V 4.51.6.0). Only if the RI difference between the tested 1st D value and the literature value was less than 20 were the volatile compounds used for further analysis.

### 2.5. Data Processing Method

Partial least squares discriminant analysis (PLS-DA) was performed using Simca-P 13.0 software (Umetrics AB, Umea, Sweden) using the Pareto scaling mode. The significance of the differences in the pairwise comparisons and the whole comparison were calculated via one-way analysis of variance (ANOVA, New Providence, NJ, USA) and a Tukey s-b(K) test using PASW Statistics software (Version 18.0, Chicago, IL, USA), respectively. A heat-map was generated using MultiExperiment Viewer 4.8.1 (Oracle Corporation, Redwood, CA, USA) after the data were UV-scaled.

## 3. Results and Discussion

### 3.1. Effect of Re-Rolling on Black Tea Aroma Quality

**Figure 2:**
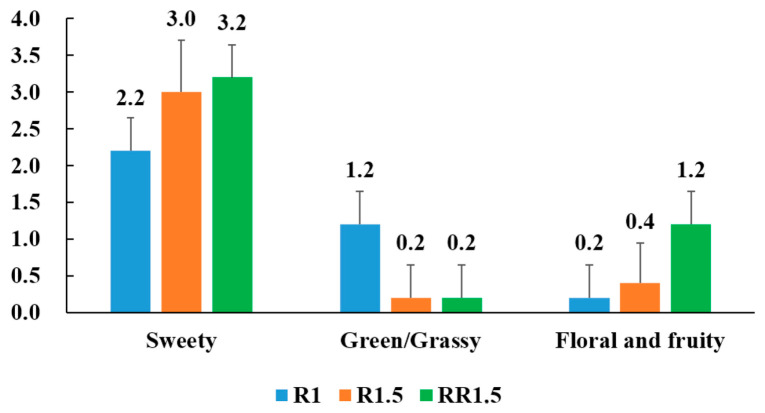
Sensory evaluation of black teas. The numbers represent the average odor intensity.

As shown in Figure 2, the scent profiles of the three black teas were largely different. The intensity of the sweet scent in R1.5 (3.0) and RR1.5 (3.2) was stronger than that in R1 (2.2). Additionally, R1 (1.2) had a slight green scent, which was slightly presented in R1.5 (0.2) and RR1.5 (0.2). An obvious floral and fruity scent was exclusively released by RR1.5 (1.2). In our previous study, it was found that tea leaves presented obvious floral and fruity scents after fermenting for 1–3 h, which gradually decreased along with the extension of fermentation [8]. In this study, the fermentation time was 5 h. At this point, the floral and fruity scents had disappeared in fermented tea leaves, thus showing that R1 and R1.5 black tea possess less floral and fruity scents. Unlike R1 and R1.5, RR1.5 black tea undergoes re-rolling treatment during the middle of the fermenting period. After re-rolling, the biochemical conditions in tea leaves are remodeled and thus create a novel occasion for generating floral and fruity scents in the later fermenting period. Actually, obvious floral and fruity scents were smelled in the RR1.5 fermenting tea leaves during the latter fermenting period. This is maybe the reason for RR1.5 black tea presenting obvious floral and fruity scents.

Black teas have multiple fragrances, such as sweet, honey, floral, fruity, and roasted odors. Among them, the sweet scent is a typical aroma class for most Congou black teas, while floral and fruity aromas are more advanced odors compared with the sweet scent for black tea, and give black tea a better aroma quality [8]. A green scent is generally considered a negative odor class for black tea [9]. Thus, the aroma quality of RR1.5 is better than that of R1.5, and which is better than that of R1. The results of the equivalent quantification of aroma indicated that re-rolling treatment was beneficial to improving the aroma quality of Congou black tea.

### 3.2. Integral Profiles of Volatile Compounds

**Figure 3:**
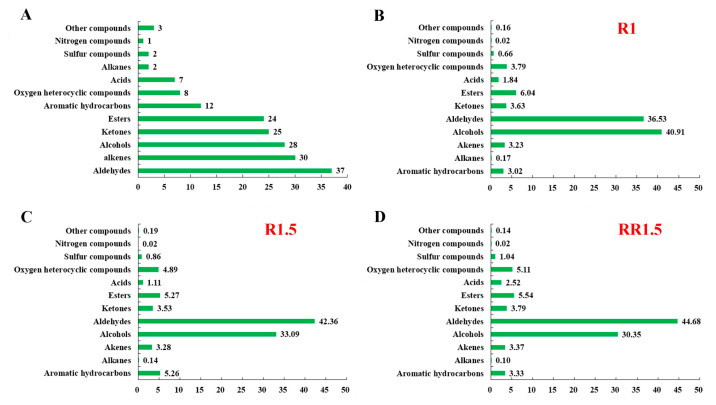
Number of different kinds of volatile compounds and their proportions in the three black teas. (**A**) Number of different kinds of volatile compounds; (**B**–**D**) proportions of different kinds of volatile compounds in R1, R1.5, and RR1.5, respectively.

Overall, a total of 316 volatile compounds were detected after peak alignment (the signal-to-noise ratio was set to 20). Among them, 179 volatile compounds were identified as reliable volatiles after RI comparison and standard verification, and they were used for further analysis (Table S1). According to their chemical structures, these volatile compounds consisted of 37 aldehydes, 30 alkenes, 28 alcohols, 25 ketones, 24 esters, 12 aromatic hydrocarbons, 8 oxygen heterocyclic compounds, 7 acids, 2 alkanes, 2 sulfur compounds, 1 nitrogen compound, and 3 other compounds (Figure 3A and Table S1). Aldehydes and alcohols were the main volatile compounds, reaching 77.44%, 75.45%, and 75.03% in the R1, R1.5, and RR1.5 black teas, respectively (Figure 3B–D). Compared with R1, the content of alcohols reduced to 33.09% (R1.5) and 30.35% (RR1.5), while the content of aldehydes increased to 42.36% (R1.5) and 44.68% (RR1.5), implying that re-rolling treatment is beneficial to increasing the content of aldehydes and reduce the content of alcohols. In addition, the levels of ketones, esters, acids, and oxygen heterocyclic compounds also showed obvious differences among R1, R1.5, and RR1.5 (Figure 3B–D).

### 3.3. Effect of Re-Rolling Treatment on Volatile Compounds of Black Tea

To better understand the effect of re-rolling treatment on black tea aroma quality, differential volatile compounds among R1, R1.5, and RR1.5 were screened and compared using multivariate statistical analysis.

#### 3.3.1. Effect of Re-Rolling Treatment on the Overall Aroma Difference

**Figure 4:**
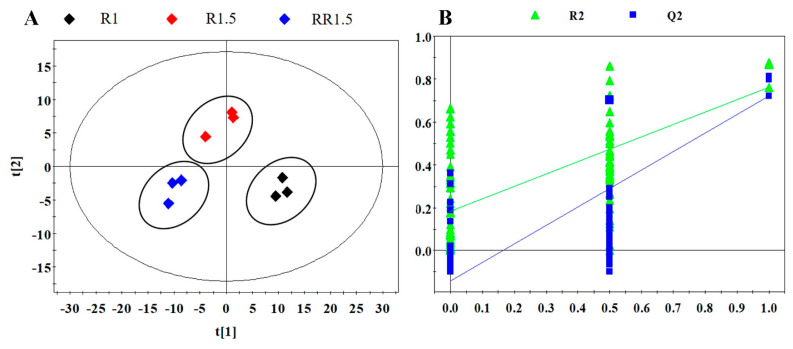
Multivariate analysis of aroma components of the three black teas. (**A**) PLS-DA score plot (R^2^X = 0.629, R^2^Y = 0.939, Q^2^ = 0.758); (**B**) cross validation model (R^2^ = 0.183, Q^2^ = −0.142).

PLS-DA was used to analyze the overall aroma difference of the three black teas based on above 179 volatile components. As shown in Figure 4A, the tea samples were clearly separated into three regions, indicating that there were obvious differences in aroma among R1, R1.5, and RR1.5 and that re-rolling treatment significantly altered the aroma components of black tea. The cross-validation analysis of the above PLS-DA model showed that this model was reliable (Figure 4B, R^2^ = 0.183, Q^2^ = −0.142).

#### 3.3.2. Pairwise Comparisons of Volatile Compounds among the Three Black Teas

**Figure 5:**
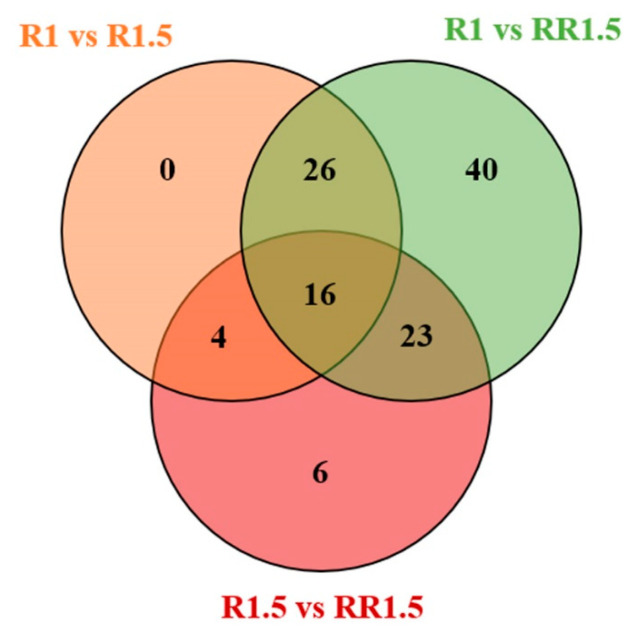
Venn diagram of differential volatile compounds in different comparison groups.

Differential volatile compounds between any two tea samples were screened via ANOVA (*p* < 0.05). There were 46, 105, and 48 differential volatile compounds in the R1 vs. R1.5, R1 vs. RR1.5, and R1.5 vs. RR1.5 groups, respectively; and among them, 16 volatile compounds showed statistical differences in all three comparison groups (Figure 5 and Tables S2–S4). For these differential volatile compounds, 35, 80, and 32 volatile compounds were significantly increased, and 11, 25, and 16 volatile compounds showed reverse changes in the R1 vs. R1.5, R1 vs. RR1.5, and R1.5 vs. RR1.5 groups, respectively (Tables S2–S4). During the fermentation period, volatile compounds were derived from the degradation of fatty acids, amino acids, carotenoids, and GBVs [7,20], which were triggered by the rolling step [21,22]. It was reported that biochemistry reactions during the fermentation period were greatly accelerated with the extension of rolling time [22]. The rolling time of R1.5 and RR1.5 was longer than that of R1, which improved the degradation of aroma precursors and thus increased the concentrations of volatile compounds in R1.5 and RR1.5 black teas. This explains why the numbers of increased volatile compounds were higher than that decreased volatile compounds in the R1 vs. R1.5 and R1 vs. RR1.5 groups.

**Figure 6:**
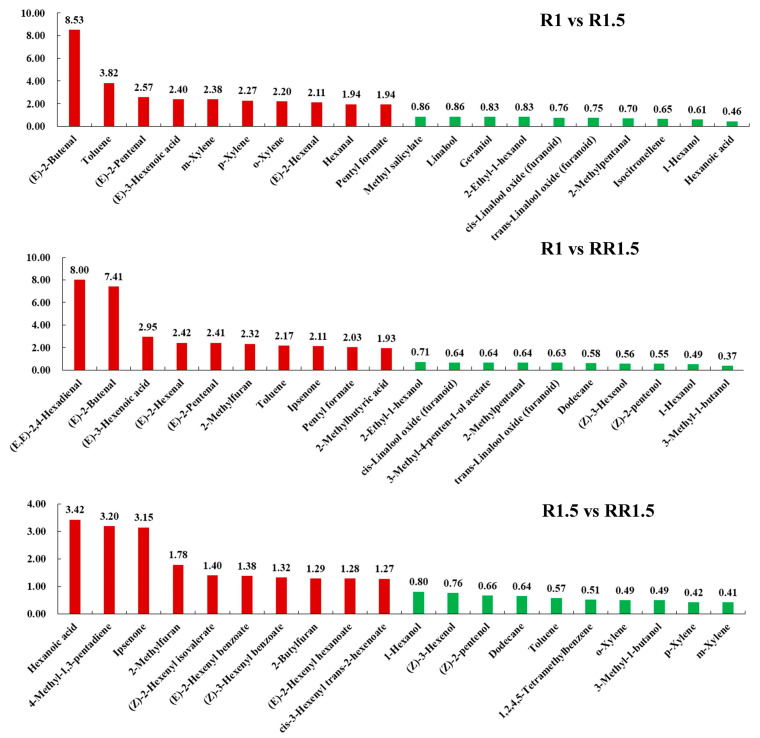
Top fold-changed non-volatile compounds. The numbers represent fold changes. Red color means increasing; green color means decreasing.

To better investigate the differences in volatile compounds among the R1, R1.5, and RR1.5 black teas, we screened and compared the top differential compounds (TDCs) with high fold changes (FCs). Compared with R1, the up-regulated TDCs in R1.5 were mainly aldehydes and aromatic hydrocarbons, including (E)-2-butenal, toluene, (E)-2-pentenal, (E)-3-hexenoic acid, m-xylene, and (E)-2-hexenal amongst others, while the down-regulated TDCs were mainly alcohols and other compounds, including hexanoic acid, 1-hexanol, isocitronellene, 2-methylpentanal, trans-linalool oxide (furanoid), and cis-linalool oxide (furanoid) amongst others (Figure 6). Compared with R1, the up-regulated TDCs in RR1.5 were also mainly aldehydes, including (E,E)-2,4-hexadienal, (E)-2-butenal, (E)-3-hexenoic acid, (E)-2-hexenal, (E)-2-pentenal, ipsenone, and α-ionone amongst others, while the down-regulated TDCs were mainly alcohols, including 3-methyl-1-butanol, 1-hexanol, (Z)-2-pentenol, (Z)-3-hexenol, dodecane, and trans-linalool oxide (furanoid) amongst others (Figure 6). It is worth noting that the FCs of increased TDCs were higher than those of decreased TDCs in the above comparison groups. This indicated that R1.5 and RR1.5 had a higher aroma intensity compared with R1. Compared with R1.5, the up-regulated TDCs in RR1.5 were mainly esters, ketones, and aldehydes, such as hexanoic acid, 4-methyl-1,3-pentadiene, ipsenone, (Z)-2-hexenyl isovalerate, (E)-2-hexenyl benzoate, and (Z)-3-hexenyl benzoate amongst others, while the down-regulated TDCs were aromatic hydrocarbons and alcohols, including m-xylene, p-xylene, 3-methyl-1-butanol, (Z)-2-pentenol, and 1-hexanol amongst others (Figure 6).

### 3.4. Overall Comparison of Aroma Quality and Volatile Compounds among the Three Black Teas

**Figure 7:**
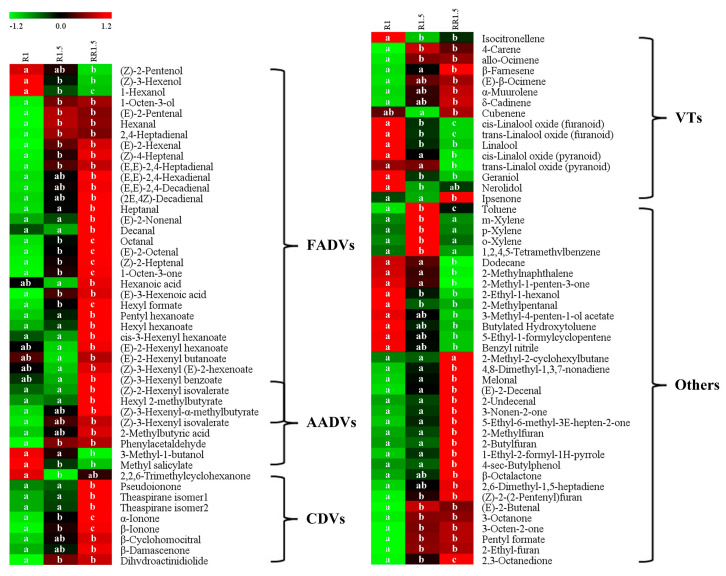
Heat-map of the levels of differential nonvolatile compounds. FADVs: fatty acid-derived volatiles; AADVs: amino acid-derived volatiles; CDVs: carotenoid-derived volatiles; VTs: volatile terpenoids. The data were UV-scaled. a, b, c: *p* < 0.05 for the changes with different letters (Tukey s-b (K) test).

The differential volatile compounds among the three black teas were screened via the Tukey s-b (K) test (*p* < 0.05). In total, 97 volatile compounds showed statistical differences, of which, 61 aroma components belonged to FADVs, AADVs, CDVs, and VTs (Figure 7).

#### 3.4.1. Differential FADVs

FADVs are major volatile secondary metabolites present in tea leaves that influence the aroma quality of teas [23]. In this study, 34 FADVs showed statistical differences among the R1, R1.5, and RR1.5 black teas (Figure 7). They mainly consisted of aldehydes and esters, followed by several alcohols, ketones, and acids. Most aldehydes, such as octanal, (E)-2-octenal, (Z)-2-heptenal, heptanal, (E)-2-hexenal, (E,E)-2,4-hexadienal, and (E)-2-nonenal, showed gradients increasing from R1 to RR1.5. The levels of 1-hexanol, (Z)-3-hexenol, and (Z)-2-pentenol were significantly decreased from R1 to RR1.5, while 1-octen-3-ol showed the opposite trend. In our previous study, we found that the levels of alcoholic FADVs significantly decreased along with fermenting time, while the levels of aldehydic FADVs displayed reverse change trends [8]. This implies that fermentation is beneficial to increasing the contents of aldehydic FADVs and reducing the contents of alcoholic FADVs. The score plot of PLS-DA showed that the fermentation degrees increased from R1 to RR1.5 (Figure 4). Thus, RR1.5 had the lowest alcoholic and highest aldehydic FADVs. Although ester FADVs showed two distribution patterns in R1 and R1.5, all esters, including hexyl formate, pentyl hexanoate, hexyl hexanoate, cis-3-hexenyl hexanoate, (E)-2-hexenyl hexanoate, (E)-2-hexenyl butanoate, (Z)-3-hexenyl (E)-2-hexenoate, (Z)-3-hexenyl benzoate, (Z)-2-hexenyl isovalerate, hexyl 2-methylbutyrate, (Z)-3-hexenyl-α-methylbutyrate, and (Z)-3-hexenyl isovalerate amongst others, showed the highest contents in RR1.5. In addition, the levels of 1-octen-3-one, hexanoic acid and (E)-3-hexenoic acid were also highest in RR1.5 black tea.

Saturated and unsaturated alcoholic and aldehydic FADVs are important contributors to fresh, green, or grassy tea aromas [7,24]. R1.5 and RR1.5 black teas had significantly more aldehydic FADVs than R1 black tea (Figure 7), which seems to contradict that the intensity of the green/grassy odor of R1.5 and RR1.5 black teas was lower than that of R1 black tea (Figure 2). Volatile components have synergistical, additive, or suppressive effects among each other according to unpredictable rules [25]. This seemingly contradictory result indicates that R1.5 and RR1.5 black teas have more sweet, floral, or fruity volatile compounds than that R1 black tea. Unlike alcohols and aldehydes, most ester FADVs generally display pleasant fruity, floral, and/or fresh scents [26,27]. For instance, hexyl formate, cis-3-hexenyl hexanoate, (Z)-3-hexenyl benzoate, and hexyl hexanoate present sweet, floral, and fruity scents [28,29,30,31]; (Z)-3-hexenyl isovalerate and (Z)-3-hexenyl-α-methylbutyrate present fresh, clean, sweet, and floral scents [29,32,33]. In addition, hexanoic acid is a sweet volatile compound [34]. Hexanoic acid and (Z)-3-hexenyl benzoate are increased TDCs in the R1.5 vs. the RR1.5 group (Figure 6). Higher levels of ester FADVs and hexanoic acid will improve the intensity of floral and fruity odors and may be an important factor for the floral and fruity scents appearing in RR1.5 black tea.

#### 3.4.2. Differential AADVs

AADVs comprise phenylpropanoid/benzenoid volatiles and branched-chain amino acid-derived volatiles. In the present study, 20 AADVs were detected, and 9 AADVs showed statistical differences among R1, R1.5, and RR1.5 (Figure 7). Most AADVs, including (Z)-3-hexenyl benzoate, (Z)-2-hexenyl isovalerate, hexyl 2-methylbutyrate, (Z)-3-hexenyl-α-methylbutyrate, (Z)-3-hexenyl isovalerate, 2-methylbutyric acid, and phenylacetaldehyde, showed the highest contents in RR1.5. In addition, the levels of benzyl alcohol also showed a large increase from R1 to RR1.5 (Table S1). Contrarily, the levels of 3-methyl-1-butanol and methyl salicylate were significantly decreased from R1 to RR1.5. (Z)-3-hexenyl benzoate, phenylacetaldehyde, benzyl alcohol, (Z)-3-hexenyl isovalerate, and (Z)-3-hexenyl-α-methylbutyrate present delightful floral, fruity, sweet, and fresh odors, while methyl salicylate generally presents minty and/or wintergreen-like scents [28,29,33]. The changes in the levels of AADVs were deemed to play an important role in the formation of floral and fruity scents in RR1.5 black tea (Figure 2).

#### 3.4.3. Differential CDVs

CDVs derive from the enzymatic oxidative, photo-oxidation, or thermal degradation of carotenoids, including β-carotene, α-carotene, phytoene, and lycopene amongst others, during tea processing, especially the fermentation and drying steps [7,35]. In our study, 16 CDVs were detected and 9 CDVs were significantly different (Table S1 and Figure 7). Compared with R1, except for 2,2,6-trimethylcyclohexanone, the levels of β-ionone, α-ionone, and dihydroactinidiolide in R1.5 showed significant increases (*p* < 0.05, Figure 7), and the levels of β-damascenone, β-cyclohomocitral, pseudoionone, and theaspirane were also obviously increased (*p* > 0.05, Figure 7). Carotenoids are synthesized and stored in chloroplasts and chromoplasts in plants [36]. There are few kinds of CDVs and their concentrations are very low in fresh tea leaves [6]. After rolling, the cell structure of the tea leaf is destroyed, which is beneficial to promoting contact between enzymes and substrates, and thus, the production of CDVs. During the rolling and fermenting steps, the oxidized degradation of carotenoids into CDVs includes carotenoid cleavage dioxygenases (CCDs)-induced enzymatic [20] and lipoxygenase (LOX)-induced co-enzymatic degradation [37]. As the rolling time of R1.5 was more than that of R1, the damage degree of the cell structure of R1.5 was surely more than that of R1. This is beneficial to accelerating the oxidized degradation of carotenoids into CDVs and thus leads to R1.5 having more CDVs compared with R1. It is interesting that the levels of all CDVs in RR1.5 were further largely increased compared with R1.5 (Figure 7). This implies that re-rolling treatment accelerates the oxidized degradation of carotenoids. Compared with R1.5, most FADVs in RR1.5 showed higher content (Figure 7), indicating that the degradation of lipids in RR1.5 was more intense than that in R1.5. In turn, the oxidized degradation of lipids accelerated the degradation of carotenoids, and thus, the production of CDVs. During the later fermentation stage, the exhaustion of substrates surrounding enzymes and the inhibition of reaction products on enzymes will suppress the production of volatile compounds. Re-rolling treatment reconstructed the biochemical conditions in tea leaves in the late fermentation period. This reconstitution will renewably allow enzymes to make sufficient contact with their substrates and thus accelerate the production of volatile compounds, such as CDVs and FADVs.

CDVs, including β-ionone, α-ionone, β-damascenone, dihydroactinidiolide, theaspirane, geranylacetone, etc., generally present pleasant floral, fruity, sweet, cream-like, or woody scents [7,32,38]. In addition, CDVs generally have markedly lower odor thresholds compared with FADVs, AADVs, and VTs [7,39]. Many CDVs, such as β-ionone, geranyl acetone, α-ionone, β-damascenone, dihydroactinidiolide, safranal, theaspirane, etc., play vital roles in deciding the aroma quality of black tea [38,40]. Therefore, the significant increase in the levels of CDVs was considered to substantially contribute to the formation of floral and fruity scents in RR1.5 black tea.

#### 3.4.4. Differential VTs

VTs are widely distributed and important aroma components in various plants. In our study, 16 VTs, mainly alkenes and alcohols, were shown to be significantly different among the three black teas (Figure 7). The levels of most alkene VTs, such as β-farnesene, (E)-β-ocimene, α-muurolene, and δ-cadinene, were gradually increased from R1 to RR1.5, while the levels of most alcoholic VTs, such as linalool, cis-linalool oxide (furanoid), trans-linalool oxide (furanoid), geraniol, etc., showed reverse change trends. These alcoholic VTs were decreased TDCs in R1.5 and RR1.5 compared with R1 (Figure 6). Most VTs, especially linalool, linalool oxides, geraniol, and nerolidol, are vital floral and fruity flavor components in teas [7,29,41]. Owing to the fact that the decreased degree of alcoholic VTs was largely more than that of alkene VTs (Tables S2–S4), it can be perceived that the alteration in the levels of VTs was not beneficial to the formation of floral and fruity scents in RR1.5 black tea.

#### 3.4.5. Other Differential Compounds

In addition, 34 other compounds also showed statistical differences among R1, R1.5, and RR1.5, and they showed four distribution patterns (Figure 7).

The levels of 2-methyl-1-penten-3-one, 2-ethyl-1-hexanol, 2-methylpentanal, 3-methyl-4-penten-1-ol acetate, benzyl nitrile, etc., were the most abundant in R1. Benzyl nitrile is an aromatic volatile compound and has been reported as one of the main aroma-active compounds in Keemun black tea [10]. However, benzyl nitrile is a toxic volatile compound commonly induced by pest insects in tea leaves [42]. From a safety standpoint, the decline in benzyl nitrile content has a positive effect on black tea quality.

The levels of toluene, m-xylene, p-xylene, o-xylene, and 1,2,4,5-tetramethylbenzene in R1.5 were higher than those in R1 and RR1.5. These compounds are all aromatic hydrocarbons. p-Xylene and o-xylene are sweet volatile compounds [40].

The levels of melonal, (E)-2-decenal, 2-undecenal, 3-nonen-2-one, 5-ethyl-6-methyl-3E-hepten-2-one, 1-ethyl-2-formyl-1H-pyrrole, β-octalactone, etc., were the most abundant in RR1.5. Several of them present pleasant odors and have a substantial influence on tea aroma. Melonal presents an intense watermelon- and cucumber-like fruity aroma [43]. 5-Ethyl-6-methyl-3E-hepten-2-one presents a fresh scent [29], and 1-ethyl-2-formyl-1H-pyrrole presents a roasted scent and is an aroma-active compound in black teas [2,40]. (E)-2-decenal presents an orange scent and is an aroma-active compound in black tea [34]. β-Octalactone is related to peach-like and creamy scents and has been identified as an aroma-active compound in Jinmudan black tea [44]. The increase in their contents is beneficial to improving the aroma quality of black tea.

The levels of 2,6-dimethyl-1,5-heptadiene, 3-octanone, (E)-2-butenal, and 3-octen-2-one, etc., in R1.5 and RR1.5 were significantly higher than those in R1. 3-Octanone and 3-octen-2-one contribute a mushroom-like aroma [45,46].

## 4. Conclusions

In this study, the effects of re-rolling treatment on Congou black tea aroma were investigated using the methods of the equivalent quantification of aroma and HS-SPME-GC-MS. Re-rolling treatment improved the aroma quality of Congou black tea by conferring upon it floral and fruity scents. In total, 179 volatile compounds were identified, of which 97 volatile compounds showed statistical differences. Re-rolling treatment significantly reduced the levels of alcoholic FADVs and VTs, but increased the levels of aldehydic and ester FADVs, most AADVs, CDVs, alkene VTs, and some other important volatile compounds. Based on the odor characteristics and FCs of differential volatile compounds, hexanoic acid, hexyl formate, cis-3-hexenyl hexanoate, (Z)-3-hexenyl benzoate, and hexyl hexanoate, phenylacetaldehyde, benzyl alcohol, β-ionone, α-ionone, dihydroactinidiolide, ipsenone, β-farnesene, β-octalactone, melonal, etc., were considered potential key odorants responsible for the formation of floral and fruity scents in RR1.5 black tea. In summary, this study provides a novel and simple processing technology to improve the aroma quality of small-leaf Congou black tea.

## Data Availability

The data used to support the findings of this study can be made available by the corresponding author upon request.

## Supplementary Materials

The following supporting information can be downloaded at: https://www.mdpi.com/article/10.3390/foods12193702/s1, Table S1: Identified volatile compounds and their peak areas in different black tea samples, Table S2: The fold changes (FC) and *p*-values of differential volatile compounds in the R1 vs. R1.5 group, Table S3: The fold changes (FC) and *p*-values of differential volatile compounds in the R1 vs. RR1.5 group, Table S4: The fold changes (FC) and *p*-values of differential volatile compounds in the R1.5 vs. RR1.5 group.
