# Supplementary material for: Re-Rolling Treatment in the Fermentation Process Improves the Aroma Quality of Black Tea
Source: Foods. 2023 Oct 9;12(19):3702. doi: 10.3390/foods12193702 (PMC10572315; doi:10.3390/foods12193702)
Supplement: Supplementary file 1 [file foods-12-03702-s001.zip › foods-2646598-supplementary.pdf]

Re-Rolling Treatment in the Fermentation Process Improves the Aroma Quality of Black Tea

Qincao Chen [1,†], Penghui Yu [2,†], Ziyi Li [1], Yuhang Wang [1], Yafang Liu [1], Yin Zhu [3,*] and Haihui Fu [1,*]

[1] College of Agriculture, Jiangxi Agricultural University, No. 1101 Zhimin Avenue, Nanchang 330045, China; chenqincao@jxau.edu.cn (Q.C.); 18931077210@163.com (Z.L.); carti18942330763@163.com (Y.W.); lyf2805101190@163.com (Y.L.)
[2] Tea Research Institute, Hunan Academy of Agricultural Sciences, No. 702 Yuanda 2nd Road, Changsha 410125, China; hncys-yupenghui@hunaas.cn
[3] Tea Research Institute, Chinese Academy of Agricultural Sciences, No. 9 Meiling South Road, Hangzhou 310008, China
* Correspondence: zhuy_scu@tricaas.com (Y.Z.); fuhh@jxau.edu.cn (H.F.)
† These authors contributed equally to this work.

Table S1. Identified volatile compounds and their peak areas in different black tea samples

NO.	Peak	CAS	Tested RI	Referred RI	Category	R1	R1.5	RR1.5	*P*-value
	**Alkanes**								
1	Dodecane	112-40-3	1199	1200		571422±72491a	516537±55944a	332790±21159b	0.004
2	Farnesane	3891-98-3	1399	1379		152825±13802a	129088±11247a	121800±24703a	0.158
	**aromatic hydrocarbons**								
3	Toluene	108-88-3	750	759		2816410±429419a	10756413±270611b	6099123±891908c	0.000
4	m-Xylene	108-38-3	854	862		699665±198166a	1666336±152076b	687241±37142a	0.000
5	p-Xylene	106-42-3	861	866		682294±517206a	1552080±136006b	648676±64647a	0.020
6	o-Xylene	95-47-6	886	889		1484518±939061a	3271697±271018b	1616801±80602a	0.015
7	Cumene	98-82-8	920	914		698399±502369a	901719±351893a	441779±64034a	0.350
8	o-Cymene	527-84-4	1022	1021		6233602±56916a	6134748±514593a	5928498±191541a	0.526
9	m-Cymenene	1124-20-5	1087	1084		353720±4521a	347624±33323a	311105±12002a	0.088
10	1,2,4,5-Tetramethylbenzene	95-93-2	1116	1131		126973±2903a	226179±32283b	114747±1107a	0.001
11	2-Methylnaphthalene	91-57-6	1285	1299		11646±1261a	10763±524a	8640±210b	0.009
12	1,1,6-Trimethyl-1,2-dihydronaphthalene	30364-38-6	1348	1355		7303±1846a	8211±2935a	7927±832a	0.862
13	Butylated Hydroxytoluene	128-37-0	1511	1517		74164±7684a	60618±9366ab	53082±2926b	0.030
14	Cadalin	483-78-3	1672	1675		7109±2552a	8172±630a	8181±719a	0.652
	**Alkenes**								
15	4-Methyl-1,3-pentadiene	926-56-7	596	609		90208±86270a	27340±6647a	87358±30493a	0.329
16	2,6-Dimethyl-1,5-heptadiene	6709-39-3	882	882		45968±2364a	50456±2200ab	54445±3533b	0.026
17	Isocitronellene	85006-04-8	914	926	VT	123238±15707a	79885±5668b	94438±6696b	0.006
18	3,5,5-Trimethyl-2-hexene	26456-76-8	973	985		273924±14054a	263045±43970a	301577±46951a	0.485
19	4-Carene	29050-33-7	988	1009	VT	25681±2414a	32874±977b	31415±245b	0.003
20	α-Fellandrene	99-83-2	1001	1000	VT	1155623±45991a	1254071±140773a	1295110±71172a	0.258
21	α-Terpinene	99-86-5	1013	1003	VT	90403±3963a	104406±13523a	108061±8336a	0.130
22	γ-Terpinene	99-85-4	1015	1030	VT	94351±25955a	66720±10220a	68117±23794a	0.273
23	Limonene	138-86-3	1025	1030	VT	6015561±338482a	6653925±782289a	6824900±54493a	0.187

24	5-Ethyl-1-formylcyclopentene	36431-60-4	1028	1026		149667±7057a	135392±10480ab	123917±7423b	0.027
25	(E)-β-Ocimene	3779-61-1	1039	1040	VT	264748±14290a	324590±41659ab	333873±14917b	0.039
26	β-Ocimene	13877-91-3	1048	1046	VT	2471437±203674a	2933632±454402a	3088188±64831a	0.091
27	Terpinolene	586-62-9	1085	1088	VT	503357±14271a	536388±64080a	535063±22311a	0.553
28	4,8-Dimethyl-1,3,7-nonadiene	19945-61-0	1117	1117		138444±5321a	154689±9361a	180711±16518b	0.011
29	1,3,8-p-Menthatriene	18368-95-1	1121	1119	VT	71978±4575a	81911±6425a	83192±3732a	0.065
30	allo-Ocimene	7216-56-0	1129	1131	VT	275826±9813a	320363±26641b	322809±13332b	0.033
31	(4E,6E)-Allocimene	3016-19-1	1140	1131	VT	199404±24486a	231433±36347a	243094±9537a	0.182
32	α-Terpineol	10482-56-1	1188	1187	VT	488758±26545a	488022±54704a	466501±33293a	0.752
33	α-Cubebene	17699-14-8	1347	1351	VT	106810±9320a	113392±10121a	129116±7193a	0.055
34	cis-β-Farnesene	28973-97-9	1415	1428	VT	55570±1849a	60934±17586a	61038±6868a	0.790
35	Humulene	6753-98-6	1449	1452	VT	108653±4588a	121781±28889a	126050±3393a	0.472
36	β-Farnesene*	77129-48-7	1456	1455	VT	36568±1077a	45829±2512a	56132±7162b	0.005
37	Germacrene D	23986-74-5	1473	1480	VT	13314±3069a	16268±2590a	16145±4061a	0.504
38	α-Muurolene	10208-80-7	1497	1497	VT	65453±1218a	71938±5776ab	74723±1870b	0.047
39	α-Farnesene	502-61-4	1507	1505	VT/CDV	23833±2092a	22426±3778a	25286±2003a	0.487
40	Calamenene	483-77-2	1520	1527	VT	874274±44186a	968173±58264a	981115±30230a	0.055
41	δ-Cadinene	483-76-1	1521	1524	VT	300089±12849a	342430±32581ab	362303±12535b	0.031
42	Cubenene	29837-12-5	1529	1513	VT	21586±1177ab	18042±1942a	22318±1590b	0.036
43	α-Calacorene	21391-99-1	1540	1542	VT	12719±705a	11706±1492a	13939±1731a	0.220
44	Neophytadiene	504-96-1	1842	1838	VT	7740±1521a	7954±1228a	9606±2028a	0.369
	**Alcohols**								
45	3-Methyl-1-butanol	123-51-3	720	726	AADV	57719±9406a	43794±3609a	21433±6936b	0.002
46	1-Pentanol	71-41-0	753	763	FADV	1490178±270186a	1588054±111403a	1280679±162469a	0.218
47	(Z)-2-pentenol	1576-95-0	760	769	FADV	2114798±477328a	1772285±131585ab	1169151±198488b	0.025
48	(Z)-3-Hexenol*	928-96-1	852	851	FADV	11115041±1864685a	8145185±944450b	6195826±616019b	0.009
49	(E)-2-Hexenol	928-95-0	863	852	FADV	1830113±660838a	1251113±349826a	999029±129982a	0.136
50	1-Hexanol*	111-27-3	866	865	FADV	9765593±247595a	5994946±371294b	4794893±315483c	0.000

51	Linalool-3,7-oxide	7392-19-0	969	971	VT	110312±9670a	115730±16036a	115716±6114a	0.804
52	1-Heptanol	111-70-6	971	967	FADV	896333±50030a	907862±87858a	892328±58942a	0.958
53	1-Octen-3-ol*	3391-86-4	979	986	FADV	4241123±233661a	5071255±306378b	5165420±340258b	0.016
54	cis-Anhydrolinalool	54750-69-5	1005	1007	VT	53372±4269a	53793±11191a	57570±3529a	0.746
55	2-Ethyl-1-hexanol	104-76-7	1030	1031		1684512±78834a	1392400±157246b	1199253±42531b	0.004
56	Benzyl alcohol	100-51-6	1034	1045	AADV	3252497±1847742a	7717482±5168190a	11074733±1520219a	0.070
57	cis-Linalool oxide (furanoid)*	5989-33-3	1071	1064	VT	6806606±183026a	5154254±192801b	4333389±229804c	0.000
58	1-Octanol	111-87-5	1072	1068	FADV	1421472±59098a	1499777±115702a	1564331±62611a	0.190
59	trans-Linalool oxide (furanoid)*	34995-77-2	1087	1087	VT	11633708±461899a	8748930±310685b	7356637±379121c	0.000
60	Linalool*	78-70-6	1102	1098	VT	51622770±2601166a	44142686±3478152b	40246694±1110780b	0.005
61	Hotrienol	29957-43-5	1105	1108	VT	8464733±419660a	8168068±1051234a	8705896±259738a	0.638
62	Phenylethyl Alcohol	60-12-8	1111	1121	AADV	29285710±1739404a	28603892±1868901a	27435221±2225251a	0.539
63	(Z)-3-Nonen-1-ol	10340-23-5	1154	1156		167605±11468a	165627±25718a	184931±9776a	0.380
64	cis-Linalol oxide (pyranoid)*	14009-71-3	1168	1173	VT	1042967±70189a	909868±58601a	762209±56684b	0.004
65	1-Nonanol	143-08-8	1172	1186	FADV	861614±24686a	810378±59477a	827536±65323a	0.521
66	trans-Linalol oxide (pyranoid)*	39028-58-5	1168	1171	VT	2515503±87601a	2488640±233195a	2131985±26710b	0.031
67	4-Terpinenol	562-74-3	1174	1172	VT	38231±4249a	33682±6419a	32429±1397a	0.323
68	γ-Isogeraniol	13066-51-8	1218	1222	VT	694495±32837a	656186±59330a	672353±166117a	0.903
69	Nerol	106-25-2	1227	1229	VT	1047911±65944a	886533±121827a	919345±85561a	0.165
70	Geraniol*	106-24-1	1256	1259	VT	25769711±1090887a	21431450±1657520b	19606191±471063b	0.002
71	Nerolidol*	40716-66-3	1563	1565	VT	488503±72886a	361807±30178b	395544±22759ab	0.040
72	Phytol	150-86-7	2115	2116		27732±7796a	26763±5835a	24144±3967a	0.765
	**Aldehydes**								
73	2-Methylpropanal	78-84-2	533	537	AADV	1557107±1015817a	1431389±624624a	2729304±1363561a	0.316
74	3-Methylbutanal	590-86-3	611	649	AADV	6569875±5107068a	6643096±4374612a	8104601±240058a	0.865
75	2-Methylbutanal	96-17-3	625	646	AADV	14850268±9777461a	16454979±10400634a	19861537±765478a	0.759
76	(E)-2-Butenal	123-73-9	610	615		146155±41625a	1246082±112670b	1082990±71888b	0.000
77	Pentanal	110-62-3	674	698	FADV	4762169±624255a	5662566±1779847a	6317962±591540a	0.316

78	2-Methyl-2-butenal	1115-11-3	728	730		418154±72367a	361863±57492a	347124±31195a	0.333
79	(E)-2-Pentenal	1576-87-0	740	754	FADV	875833±86954a	2252363±379855b	2113131±68104b	0.001
80	2-Methylpentanal	123-15-9	745	758		302624±30975a	211069±7622b	192307±3532b	0.001
81	Hexanal*	66-25-1	795	801	FADV	9100569±803361a	17689571±751896b	16983313±989890b	0.000
82	(E)-2-Hexenal*	6728-26-3	841	850	FADV	11485375±3319958a	24223696±1450802b	27827075±993717b	0.000
83	(Z)-4-Heptenal	6728-31-0	898	904	FADV	107774±13458a	142380±2715b	162360±14924b	0.004
84	Heptanal	111-71-7	900	903	FADV	8154933±71523a	8714515±272733a	9344194±358112b	0.004
85	(E,E)-2,4-Hexadienal*	142-83-6	908	915	FADV	25725±14564a	118903±62068ab	205824±43728b	0.008
86	(Z)-2-Heptenal	57266-86-1	954	958	FADV	790759±19360a	948708±42674b	1068360±47044c	0.000
87	Benzaldehyde	100-52-7	955	961	AADV	24805558±899084a	26175437±2309837a	26046351±1459430a	0.568
88	2,4-Heptadienal	5910-85-0	995	1007	FADV	3044277±411930a	4192485±339107b	4103731±316573b	0.014
89	Octanal*	124-13-0	1002	1004	FADV	508426±20523a	597753±43687b	714702±14643c	0.000
90	(E,E)-2,4-Heptadienal*	4313-03-5	1009	1002	FADV	6496582±512012a	7794333±558681b	8194534±469419b	0.016
91	Phenylacetaldehyde*	122-78-1	1041	1051	AADV	56823231±2044326a	66991480±1025440b	68054420±3320279b	0.002
92	Melonal*	106-72-9	1053	1044		97092±16613a	120797±1979a	152643±17645b	0.008
93	(E)-2-Octenal*	2548-87-0	1057	1063	FADV	1559265±39619a	1843570±85621b	2142355±96235c	0.000
94	(E,E)-2,4-Octadienal	30361-28-5	1109	1111	FADV	127651±6036a	132196±13234a	144056±8882a	0.190
95	α-Cyclocitral	432-24-6	1114	1123	CDV	212973±10244a	193063±13038a	202078±10374a	0.177
96	(E)-2-Nonenal*	18829-56-6	1159	1162	FADV	208979±9964a	219917±10542a	260125±21139b	0.012
97	2,4-Dimethylbenzaldehyde	15764-16-6	1171	1181		326760±7415a	307131±18876a	332756±19649a	0.212
98	Safranal	116-26-7	1196	1201	CDV	722187±36118a	703980±45905a	701820±9517a	0.737
99	Decanal	112-31-2	1205	1205	FADV	568381±28238a	548891±29861a	640998±19928b	0.012
100	1-p-Menthen-9-al	29548-14-9	1214	1217		75039±3328a	78173±7239a	81945±5198a	0.367
101	β-Cyclocitral	432-25-7	1217	1226	CDV	2038359±82487a	2154588±158494a	2294881±30805a	0.064
102	Neral	106-26-3	1240	1242	VT	88951±4489a	79587±6217a	90291±7851a	0.159
103	β-Cyclohomocitral	472-66-2	1254	1251	CDV	571796±30634a	611633±32245ab	640414±6585b	0.047
104	(E)-2-Decenal	3913-81-3	1261	1250		151559±10150a	176071±11288a	202867±13350b	0.005
105	Geranial	141-27-5	1270	1279	VT	1191126±35482a	1201364±136140a	1306873±40739a	0.256

106	α-Ethylidene-phenylacetaldehyde	4411-89-6	1270	1284		253293±7118a	241802±24602a	268121±13732a	0.236
107	(2E,4Z)-Decadienal	25152-83-4	1292	1312	FADV	114326±15077a	138565±5474ab	164699±16353b	0.010
108	(E,E)-2,4-Decadienal	25152-84-5	1314	1312	FADV	209565±31087a	268749±21704ab	314146±27444b	0.009
109	2-Undecenal	2463-77-6	1363	1368		59504±6230a	63783±4335a	78554±8102b	0.025
	**Ketones**								
110	3-Methyl-2-butanone	563-80-4	658	645		1717302±691361a	1296829±1141386a	978215±66930a	0.536
111	1-Penten-3-one	1629-58-9	659	678	FADV	356856±24534a	301826±8375a	329265±40119a	0.223
112	3-Penten-2-one	625-33-2	727	733		71464±4617a	82762±17806a	146189±50246a	0.051
113	2-Methyl-3-pentanone	565-69-5	735	742		63334±5887a	58307±3845a	58866±2934a	0.372
114	2-Methyl-1-penten-3-one	25044-01-3	755	748		66010±3329a	61692±2591a	51314±463b	0.001
115	2,3-Hexanedione	3848-24-6	774	786		214348±45273a	268128±19633a	265458±7945a	0.108
116	2-Heptanone	110-43-0	889	884	FADV	2418638±125970a	2631224±196698a	2837011±304036a	0.147
117	1-Octen-3-one*	4312-99-6	978	979	FADV	560822±43623a	701995±46764b	791889±16814c	0.001
118	2,3-Octanedione	585-25-1	985	987		1145000±121236a	1457002±58751b	1662807±8066c	0.001
119	3-Octanone	106-68-3	986	984		356980±12595a	437711±43605b	439124±28270b	0.027
120	6-Methyl-5-hepten-2-one	110-93-0	987	985	CDV	1486219±91022a	1279058±136207a	1315618±103764a	0.130
121	2,2,6-Trimethylcyclohexanone*	2408-37-9	1031	1023	CDV	2440672±29870a	2228902±81284b	2369612±6807a	0.006
122	3-Octen-2-one*	1669-44-9	1039	1037		1156988±132335a	1614664±130321b	1677405±91054b	0.003
123	γ-Caprolactone	695-06-7	1059	1056	FADV	172278±13558a	278932±65791a	375750±159874a	0.119
124	Ipsenone	19860-68-5	1066	1083	VT	90304±28821a	60674±23909a	190953±46949b	0.009
125	Isophorone	78-59-1	1118	1126	CDV	48006±5622a	50446±3257a	55158±7634a	0.369
126	3-Nonen-2-one	14309-57-0	1140	1136		153542±13899a	166551±15338a	199307±5251b	0.010
127	5-Ethyl-6-methyl-3E-hepten-2-one	57283-79-1	1146	1144		1417613±70830a	1587395±165591a	1893314±53429b	0.005
128	3-Methylacetophenone	585-74-0	1181	1192		36047±1392a	34034±4961a	35467±2299a	0.749
129	β-Damascenone*	23726-93-4	1382	1388	CDV	500084±31353a	534153±40448ab	591220±34308b	0.048
130	Pseudoionone	1604-34-8	1405	1407	CDV	22103±2582a	22627±1423a	26970±1518b	0.039
131	α-Ionone*	127-41-3	1426	1429	CDV	285101±21431a	362749±33931b	439720±29115c	0.002
132	Geranylacetone	3796-70-1	1452	1445	CDV	224779±19186a	201611±19881a	240363±27156a	0.183

133	β-Ionone*	14901-07-6	1484	1488	CDV	752448±38784a	906992±35734b	1005997±52380c	0.001
134	Dihydroactinidiolide*	17092-92-1	1523	1525	CDV	93215±3414a	121389±3309b	126872±4814b	0.000
	**Esters**								
135	Pentyl formate	638-49-3	823	827		259127±66505a	501681±12003b	525623±48440b	0.001
136	Methyl caproate	106-70-7	925	938	FADV	474851±48950a	454970±28470a	458212±17180a	0.757
137	Hexyl formate	629-33-4	928	927	FADV	109160±3118a	135800±10475b	163402±13730c	0.002
138	3-Methyl-4-penten-1-ol acetate	71487-16-6	1008	1006		657143±64274a	537279±97966ab	418209±86961b	0.037
139	Benzyl formate	104-57-4	1075	1082	AADV	96363±5282a	123776±21600a	94294±9840a	0.075
140	(Z)-3-Hexenyl isobutanoate	41519-23-7	1145	1143	FADV	134511±4709a	140098±14815a	155284±1772a	0.071
141	(E)-3-Hexenyl butanoate	53398-84-8	1187	1186	FADV	1948656±81477a	1912644±208818a	2085532±72931a	0.329
142	Methyl salicylate*	119-36-8	1191	1199	AADV	11328008±152147a	9740791±605279b	9029901±436991b	0.002
143	Hexyl butyrate	2639-63-6	1193	1184	FADV	143198±6854a	145781±10547a	165555±14466a	0.091
144	(E)-2-Hexenyl butanoate	53398-83-7	1196	1195	FADV	654350±16433ab	574413±66504a	676202±10782b	0.046
145	(Z)-3-Hexenyl-α-methylbutyrate	53398-85-9	1233	1233	FADV/AADV	3204437±160701a	3595800±284190ab	4046202±120078b	0.006
146	(Z)-3-Hexenyl isovalerate*	35154-45-1	1237	1245	FADV/AADV	605793±17831a	695421±58578ab	728231±32280b	0.023
147	Hexyl 2-methylbutyrate	10032-15-2	1238	1227	FADV/AADV	324285±18970a	330497±22082a	395996±5029b	0.004
148	(E)-2-Hexenyl isovalerate	68698-59-9	1240	1245	FADV/AADV	412003±23496a	383539±55677a	470344±17304a	0.065
149	(Z)-2-Hexenyl isovalerate		1246	1245	FADV/AADV	107795±6319a	102149±13305a	142505±7213b	0.004
150	β-Octalactone*	698-76-0	1283	1288		31772±5297a	36088±6604ab	46619±4554b	0.041
151	Pentyl hexanoate	540-07-8	1289	1288	FADV	55020±596a	60678±4689a	70004±3663b	0.005
152	Methyl geranate	1189-09-9	1324	1321	VT	219693±14783a	179694±20308a	209242±16000a	0.068
153	cis-3-Hexenyl hexanoate*	31501-11-8	1382	1380	FADV	4791843±95104a	4599033±450493a	5660186±326154b	0.015
154	Hexyl hexanoate*	6378-65-0	1387	1385	FADV	320920±12899a	338163±22918a	387174±21543b	0.015
155	(E)-2-Hexenyl hexanoate	53398-86-0	1390	1391	FADV	340337±11048ab	297972±35746a	382884±23705b	0.019
156	cis-3-Hexenyl trans-2-hexenoate	53398-87-1	1433	1401	FADV	23334±1702ab	20405±2060a	25984±2430b	0.046
157	(Z)-3-Hexenyl benzoate	25152-85-6	1568	1565	FADV/AADV	94765±7121ab	86667±4655a	114608±12901b	0.022
158	(E)-2-Hexenyl benzoate	76841-70-8	1583	1590	FADV/AADV	25787±2998a	23440±1803a	32423±5175a	0.053
	**Acids**								

159	2-Methylbutyric acid	116-53-0	877	894	AADV	15057±968a	22835±4415ab	29001±7818b	0.046
160	Hexanoic acid	142-62-1	1005	1013	FADV	5978366±1431306ab	2721934±277423a	9319470±3742702b	0.036
161	(E)-3-Hexenoic acid	1577-18-0	1023	1021	FADV	283771±5298a	680949±136019b	837363±131156b	0.002
162	(E)-2-Hexenoic acid	13419-69-7	1045	1041	FADV	22536±6206a	35017±21290a	35847±11458a	0.491
163	Levulinic acid	123-76-2	1060	1063		182445±39815a	302460±111112a	247356±52166a	0.223
164	Nonanoic acid	112-05-0	1276	1281	FADV	1070712±204888a	1053283±155982a	1078096±323599a	0.992
165	trans-Geranic acid	4698-08-2	1356	1355	VT	466567±196526a	465080±57513a	515235±99031a	0.871
	**Oxygen heterocyclic compounds**								
166	2-Methylfuran	534-22-5	569	590		30492±13922a	39746±4203a	70866±15315b	0.016
167	2-Ethyl-furan	3208-16-0	679	702		12912661±1931188a	19559460±1393131b	20008958±417828b	0.001
168	2-Butylfuran	4466-24-4	888	889		1465334±94862a	1528276±102703a	1965421±141570b	0.003
169	Pentyl-oxirane	5063-65-0	903	905		535872±24469a	352430±132486a	456470±106758a	0.156
170	(Z)-2-(2-Pentenyl)furan	70424-13-4	1000	1001		490464±6621a	571532±41344b	620708±8880b	0.002
171	2-Methyl-2-cyclohexylbutane	31797-64-5	1152	1130		160457±6909a	162754±10318a	184146±11602a	0.047
172	Theaspirane isomer1	36431-72-8	1294	1305	CDV	488258±23286a	506516±29833a	568110±2267b	0.010
173	Theaspirane isomer2	36431-72-8	1312	1305	CDV	470615±26019a	488685±26027a	542935±8385b	0.016
	**Sulfur compounds**								
174	Dimethyl sulfide	75-18-3	518	532	AADV	2799808±2469068a	4020864±343221a	4911749±1902674a	0.413
175	Dimethyl trisulfide	3658-80-8	962	972	AADV	86971±2348a	69468±13024a	70381±9858a	0.113
	**Nitrogen compounds**								
176	1-Ethyl-1H-pyrrole-2-carboxaldehyde*	2167-14-8	1046	1046		66322±4049a	75465±3374a	95074±6856b	0.001
	**Other compounds**								
177	Benzyl nitrile	140-29-4	1137	1143		242611±6819a	222717±16161ab	207489±9159b	0.026
178	4-sec-Butylphenol	99-71-8	1314	1315		32152±2180a	33346±3014a	40919±2638b	0.013
179	Caffeine	58-08-2	1846	1841		424826±182534a	624819±120658a	413377±98904a	0.191

Note: The data was shown as mean ± SD with peak area (n=3); *: the compound was confirmed by the authentic standard; a, b, c: $P < 0.05$ for the changes with different letters (Tukey s-b (K) test).

Table S2. The fold changes (FC) and *P*-values of differential volatile compounds in the R1 vs R1.5 group

	**R1**	**R1.5**	**FC**	***P*-values**
(E)-2-Butenal	146155	1246082	8.53	0.000
Toluene	2816410	10756413	3.82	0.000
(E)-2-Pentenal	875833	2252363	2.57	0.004
(E)-3-Hexenoic acid	283771	680949	2.40	0.007
m-Xylene	699665	1666336	2.38	0.003
p-Xylene	682294	1552080	2.27	0.048
o-Xylene	1484518	3271697	2.20	0.034
(E)-2-Hexenal	11485375	24223696	2.11	0.004
Hexanal	9100569	17689571	1.94	0.000
Pentyl formate	259127	501681	1.94	0.003
1,2,4,5-Tetramethylbenzene	126973	226179	1.78	0.006
2-Methylbutyric acid	15057	22835	1.52	0.041
2-Ethyl-furan	12912661	19559460	1.51	0.008
3-Octen-2-one	1156988	1614664	1.40	0.013
2,4-Heptadienal	3044277	4192485	1.38	0.020
(Z)-4-Heptenal	107774	142380	1.32	0.012
Dihydroactinidiolide	93215	121389	1.30	0.001
4-Carene	25681	32874	1.28	0.009
2,3-Octanedione	1145000	1457002	1.27	0.016
α-Ionone	285101	362749	1.27	0.029
β-Farnesene	36568	45829	1.25	0.004
1-Octen-3-one	560822	701995	1.25	0.019
Hexyl formate	109160	135800	1.24	0.013
3-Octanone	356980	437711	1.23	0.037
β-Ionone	752448	906992	1.21	0.007
(E,E)-2,4-Heptadienal	6496582	7794333	1.20	0.041
(Z)-2-Heptenal	790759	948708	1.20	0.004
1-Octen-3-ol	4241123	5071255	1.20	0.020
(E)-2-Octenal	1559265	1843570	1.18	0.006
Phenylacetaldehyde	56823231	66991480	1.18	0.002
Octanal	508426	597753	1.18	0.033
(Z)-2-(2-Pentenyl)furan	490464	571532	1.17	0.028
(E)-2-Decenal	151559	176071	1.16	0.049
1-Ethyl-1H-pyrrole-2-carboxaldehyde	66322	75465	1.14	0.040
Heptanal	8154933	8714515	1.07	0.026
2,2,6-Trimethylcyclohexanone	2440672	2228902	0.91	0.013
Methyl salicylate	11328008	9740791	0.86	0.012
Linalool	51622770	44142686	0.86	0.041
Geraniol	25769711	21431450	0.83	0.019
2-Ethyl-1-hexanol	1684512	1392400	0.83	0.045

cis-Linalool oxide (furanoid)	6806606	5154254	0.76	0.000
trans-Linalool oxide (furanoid)	11633708	8748930	0.75	0.001
2-Methylpentanal	302624	211069	0.70	0.008
Isocitronellene	123238	79885	0.65	0.011
1-Hexanol	9765593	5994946	0.61	0.000
Hexanoic acid	5978366	2721934	0.46	0.018

Note: The difference significances were calculated by ANOVA.

Table S3. The fold changes (FC) and *P*-values of differential volatile compounds in the R1 vs RR1.5 group

	**R1**	**RR1.5**	**FC**	***P*-values**
(E,E)-2,4-Hexadienal	25725	205824	8.00	0.002
(E)-2-Butenal	146155	1082990	7.41	0.000
(E)-3-Hexenoic acid	283771	837363	2.95	0.002
(E)-2-Hexenal	11485375	27827075	2.42	0.001
(E)-2-Pentenal	875833	2113131	2.41	0.000
2-Methylfuran	30492	70866	2.32	0.028
Toluene	2816410	6099123	2.17	0.005
Ipsenone	90304	190953	2.11	0.034
Pentyl formate	259127	525623	2.03	0.005
2-Methylbutyric acid	15057	29001	1.93	0.037
Hexanal	9100569	16983313	1.87	0.000
Melonal	97092	152643	1.57	0.017
2-Ethyl-furan	12912661	20008958	1.55	0.003
α-Ionone	285101	439720	1.54	0.002
β-Farnesene	36568	56132	1.53	0.009
(Z)-4-Heptenal	107774	162360	1.51	0.009
(E,E)-2,4-Decadienal	209565	314146	1.50	0.012
Hexyl formate	109160	163402	1.50	0.003
β-Octalactone	31772	46619	1.47	0.021
2,3-Octanedione	1145000	1662807	1.45	0.002
3-Octen-2-one	1156988	1677405	1.45	0.005
(2E,4Z)-Decadienal	114326	164699	1.44	0.017
1-Ethyl-1H-pyrrole-2-carboxaldehyde	66322	95074	1.43	0.003
1-Octen-3-one	560822	791889	1.41	0.001
Octanal	508426	714702	1.41	0.000
(E)-2-Octenal	1559265	2142355	1.37	0.001
Dihydroactinidiolide	93215	126872	1.36	0.001
(Z)-2-Heptenal	790759	1068360	1.35	0.001
2,4-Heptadienal	3044277	4103731	1.35	0.024
2-Butylfuran	1465334	1965421	1.34	0.007
(E)-2-Decenal	151559	202867	1.34	0.006
β-Ionone	752448	1005997	1.34	0.003

5-Ethyl-6-methyl-3E-hepten-2-one	1417613	1893314	1.34	0.001
Pentanal	4762169	6317962	1.33	0.035
(Z)-2-Hexenyl isovalerate	107795	142505	1.32	0.003
2-Undecenal	59504	78554	1.32	0.032
4,8-Dimethyl-1,3,7-nonadiene	138444	180711	1.31	0.013
3-Nonen-2-one	153542	199307	1.30	0.006
4-sec-Butylphenol	32152	40919	1.27	0.010
Pentyl hexanoate	55020	70004	1.27	0.002
(Z)-2-(2-Pentenyl)furan	490464	620708	1.27	0.000
(Z)-3-Hexenyl-α-methylbutyrate	3204437	4046202	1.26	0.002
(E,E)-2,4-Heptadienal	6496582	8194534	1.26	0.013
(E)-β-Ocimene	264748	333873	1.26	0.004
β-Ocimene	2471437	3088188	1.25	0.008
(E)-2-Nonenal	208979	260125	1.24	0.019
3-Octanone	356980	439124	1.23	0.010
4-Carene	25681	31415	1.22	0.015
Hexyl 2-methylbutyrate	324285	395996	1.22	0.003
Pseudoionone	22103	26970	1.22	0.048
(4E,6E)-Allocimene	199404	243094	1.22	0.045
1-Octen-3-ol	4241123	5165420	1.22	0.018
α-Cubebene	106810	129116	1.21	0.030
δ-Cadinene	300089	362303	1.21	0.004
Hexyl hexanoate	320920	387174	1.21	0.010
(Z)-3-Hexenyl isovalerate	605793	728231	1.20	0.005
Phenylacetaldehyde	56823231	68054420	1.20	0.008
α-Terpinene	90403	108061	1.20	0.030
β-Damascenone	500084	592318	1.18	0.025
2,6-Dimethyl-1,5-heptadiene	45968	54445	1.18	0.026
cis-3-Hexenyl hexanoate	4791843	5660186	1.18	0.011
allo-Ocimene	275826	322809	1.17	0.008
Theaspirane isomer1	488258	568110	1.16	0.004
Humulene	108653	126050	1.16	0.006
1,3,8-p-Menthatriene	71978	83192	1.16	0.030
(Z)-3-Hexenyl isobutanoate	134511	155284	1.15	0.002
Theaspirane isomer2	470615	542935	1.15	0.010
2-Methyl-2-cyclohexylbutane	160457	184146	1.15	0.038
Heptanal	8154933	9344194	1.15	0.005
α-Muurolene	65453	74723	1.14	0.002
(E)-2-Hexenyl isovalerate	412003	470344	1.14	0.026
Limonene	6015561	6824900	1.13	0.015
Decanal	568381	640998	1.13	0.022
β-Cyclocitral	2038359	2294881	1.13	0.007
(E)-2-Hexenyl hexanoate	340337	382884	1.13	0.048

Calamenene	874274	981115	1.12	0.026
α-Fellandrene	1155623	1295110	1.12	0.046
β-Cyclohomocitral	571796	640414	1.12	0.019
1-Octanol	1421472	1564331	1.10	0.045
Geranial	1191126	1306873	1.10	0.021
2,2,6-Trimethylcyclohexanone	2440672	2369612	0.97	0.016
1,2,4,5-Tetramethylbenzene	126973	114747	0.90	0.002
m-Cymenene	353720	311105	0.88	0.005
Benzyl nitrile	242611	207489	0.86	0.006
trans-Linalol oxide (pyranoid)	2515503	2131985	0.85	0.002
5-Ethyl-1-formylcyclopentene	149667	123917	0.83	0.012
Dimethyl trisulfide	86971	70381	0.81	0.047
Methyl salicylate	11328008	9029901	0.80	0.001
Linalool	51622770	40246694	0.78	0.002
2-Methyl-1-penten-3-one	66010	51314	0.78	0.002
Isocitronellene	123238	94438	0.77	0.043
Geraniol	25769711	19606191	0.76	0.001
2-Methylnaphthalene	11646	8640	0.74	0.015
cis-Linalol oxide (pyranoid)	1042967	762209	0.73	0.006
Butylated Hydroxytoluene	74164	53082	0.72	0.011
2-Ethyl-1-hexanol	1684512	1199253	0.71	0.001
cis-Linalool oxide (furanoid)	6806606	4333389	0.64	0.000
3-Methyl-4-penten-1-ol acetate	657143	418209	0.64	0.019
2-Methylpentanal	302624	192307	0.64	0.004
trans-Linalool oxide (furanoid)	11633708	7356637	0.63	0.000
Dodecane	571422	332790	0.58	0.005
(Z)-3-Hexenol	11115041	6195826	0.56	0.012
(Z)-2-pentenol	2114798	1169151	0.55	0.034
1-Hexanol	9765593	4794893	0.49	0.000
3-Methyl-1-butanol	57719	21433	0.37	0.006

Note: The difference significances were calculated by ANOVA.

Table S4. The fold changes (FC) and *P*-values of differential volatile compounds in the R1.5 vs RR1.5 group

	**R1.5**	**RR1.5**	**FC**	***P*-values**
Hexanoic acid	2721934	9319470	3.42	0.038
4-Methyl-1,3-pentadiene	27340	87358	3.20	0.029
Ipsenone	60674	190953	3.15	0.013
2-Methylfuran	39746	70866	1.78	0.027
(Z)-2-Hexenyl isovalerate	102149	142505	1.40	0.010
(E)-2-Hexenyl benzoate	23440	32423	1.38	0.047
(Z)-3-Hexenyl benzoate	86667	114608	1.32	0.024
2-Butylfuran	1528276	1965421	1.29	0.012

(E)-2-Hexenyl hexanoate	297972	382884	1.28	0.027
cis-3-Hexenyl trans-2-hexenoate	20405	25984	1.27	0.039
Melonal	120797	152643	1.26	0.036
1-Ethyl-1H-pyrrole-2-carboxaldehyde	75465	95074	1.26	0.011
Cubenene	18042	22318	1.24	0.042
cis-3-Hexenyl hexanoate	4599033	5660186	1.23	0.030
4-sec-Butylphenol	33346	40919	1.23	0.031
α-Ionone	362749	439720	1.21	0.041
Hexyl 2-methylbutyrate	330497	395996	1.20	0.007
3-Nonen-2-one	166551	199307	1.20	0.025
Octanal	597753	714702	1.20	0.012
5-Ethyl-6-methyl-3E-hepten-2-one	1587395	1893314	1.19	0.038
Pseudoionone	22627	26970	1.19	0.022
(E)-2-Nonenal	219917	260125	1.18	0.042
Isocitronellene	79885	94438	1.18	0.045
Decanal	548891	640998	1.17	0.011
(E)-2-Octenal	1843570	2142355	1.16	0.016
(E)-2-Hexenal	24223696	27827075	1.15	0.024
2,3-Octanedione	1457002	1662807	1.14	0.004
1-Octen-3-one	701995	791889	1.13	0.035
(Z)-2-Heptenal	948708	1068360	1.13	0.031
Theaspirane isomer1	506516	568110	1.12	0.023
Theaspirane isomer2	488685	542935	1.11	0.026
2,2,6-Trimethylcyclohexanone	2228902	2369612	1.06	0.040
2-Methylpentanal	211069	192307	0.91	0.018
trans-Linalool oxide (furanoid)	8748930	7356637	0.84	0.008
cis-Linalool oxide (furanoid)	5154254	4333389	0.84	0.009
cis-Linalol oxide (pyranoid)	909868	762209	0.84	0.035
2-Methyl-1-penten-3-one	61692	51314	0.83	0.002
2-Methylnaphthalene	10763	8640	0.80	0.003
1-Hexanol	5994946	4794893	0.80	0.013
(Z)-3-Hexenol	8145185	6195826	0.76	0.040
(Z)-2-Pentenol	1772285	1169151	0.66	0.012
Dodecane	516537	332790	0.64	0.006
Toluene	10756413	6099123	0.57	0.001
1,2,4,5-Tetramethylbenzene	226179	114747	0.51	0.004
o-Xylene	3271697	1616801	0.49	0.001
3-Methyl-1-butanol	43794	21433	0.49	0.008
p-Xylene	1552080	648676	0.42	0.000
m-Xylene	1666336	687241	0.41	0.000

Note: The difference significances were calculated by ANOVA.
